# Molecular and Functional Characterization of Thioredoxin 1 from Korean Rose Bitterling (*Rhodeus uyekii*)

**DOI:** 10.3390/ijms160819433

**Published:** 2015-08-17

**Authors:** Julan Kim, Ji Young Moon, Woo-Jin Kim, Dong-Gyun Kim, Bo-Hye Nam, Young-Ok Kim, Jung Youn Park, Cheul Min An, Hee Jeong Kong

**Affiliations:** Biotechnology Research Division, National Fisheries Research and Development Institute, Busan 619-705, Korea; E-Mails: tks1010@hanmail.net (J.K.); moonjy@pknu.ac.kr (J.Y.M.); wj2464@korea.kr (W.-J.K.); combikola@korea.kr (D.-G.K.); nambohye@korea.kr (B.-H.N.); yobest12@korea.kr (Y.-O.K.); genome@korea.kr (J.Y.P.); ancm@korea.kr (C.M.A.)

**Keywords:** thioredoxin, Korean rose bitterling, *Rhodues uyekii*, expression analysis, anti-oxidant, MCO assay, ROS detection

## Abstract

Thioredoxin is a multifunctional antioxidant enzyme that belongs to the reductase family. In this study, we cloned and characterized thioredoxin 1 cDNA from the Korean rose bitterling *Rhodeus uyekii* (RuTrx). The full-length RuTrx cDNA consists of 674 bp with a 324 nt open reading frame (ORF) encoding a 107 aa protein. The deduced RuTrx amino acid sequence indicated a characteristic redox active site, ^31^WCGPC^35^. Pairwise alignment revealed RuTrx amino acid identity (55.1%–83.2%) with orthologs from various species of mammalia, amphibia, fish and bird. Phylogenetic analysis was conducted to determine the evolutionary position of RuTrx. Expression analysis showed that RuTrx transcripts were present in all of the tissues examined, and was high in the hepatopancreas of *R. uyekii*. During early development, the expression of RuTrx transcripts was increased. Recombinant RuTrx protein (rRuTrx) was tested for its capacity to serve as an antioxidant enzyme using a metal-catalyzed oxidation (MCO) system. The ability of rRuTrx to protect against supercoiled DNA cleavage due to oxidative nicking increased in a dose-dependent manner. In Raw264.7 cells, Dihydroethidium (DHE) staining for ROS production indicated the antioxidant activity of rRuTrx. Together, these findings suggest that RuTrx may play a role in maintaining the redox state balance in Korean rose bitterling *R. uyekii*.

## 1. Introduction

Normal cellular metabolism is subject to moderate oxidative conditions, and reactive oxygen species (ROS) are products of normal metabolism and xenobiotic exposure. At low to moderate levels, ROS function in physiological cell processes, whereas excess ROS induces oxidative modification of cellular macromolecules, inhibits protein function, and promotes cell death [1,2]. This oxidative stress results in direct or indirect ROS-mediated damage of nucleic acids, proteins and lipids, and the accumulation of ROS elicits fatal effects, including carcinogenesis [3], neurodegeneration [4], atherosclerosis [5], diabetes [6], and aging [7,8]. Therefore, various redox systems consisting of key redox signaling components—such as thioredoxin (Trx), glutathione (GSH) and pyridine nucleotide redox couples—participate in redox homeostasis, which is critical for cellular viability and integrity [1].

Thioredoxin (Trx) is a small, 10–12 kDa protein that belongs to the reductase family and is conserved in all organisms through evolution. Trx contains the characteristic thioredoxin fold motif with a five-stranded β-sheet surrounded by four α-helices, and the highly conserved active site (Cys-Gly-Pro-Cys, CGPC) located between β-strand 2 and α-helix 2 [9]. Trx with this structure reduces disulfide bonds in several proteins and has been demonstrated to act as a hydrogen donor [10]. Trx has been classified into three distinct forms: Cytosolic Trx 1 is the most studied [11], mitochondrial Trx2 targets mitochondrial protein and has an additional 60 amino acids at the N-terminus [12], and the third isoform, SpTrx, is a novel member of the thioredoxin family that is expressed specifically in human spermatozoa [13]. Trx participates in a wide variety of cellular mechanisms, mainly redox reactions, via the reversible oxidation of its active site [14]. It is involved in processes such as cellular homeostasis, DNA replication, regulating signal transduction, proliferation of lymphoid cells and fibroblast, reducing H_2_O_2_ and scavenging free radicals, protecting cells against oxidative stress, and inhibition of apoptosis [15]. In the Trx system, oxidized Trx is reduced by thioredoxin reductase with the use of electrons supplied by NADPH [16].

Korean rose bitterling (*Rhodeus uyekii*) belongs to the Acheilognathinae subfamily of the Cyprinidae family. This common freshwater fish endemic to Korea is found in rivers that empty into the Western and Southern Sea of Korea [17]. This species has been proposed as a candidate for developing ornamental fish because of its small size and beautiful body color [18]. Genetic studies on the Korean rose bitterling have reported the complete mitochondrial genome sequence of *R. uyekii* [19], as well as the development of microsatellite markers to evaluate population genetic diversity [20]. *R. uyekii* *β-actin* gene was suggested as a promoter capable of driving constitutive transgene expression [21]. It is worth studying the stress response of *R. uyekii* since fish may need to control potential damage(s) in the water [22,23]. Antioxidant protein peroxiredoxin 1 have been cloned and characterized in *R. uyekii* [22].

To better understand the antioxidant systems in this fish, we report the identification and molecular characterization of the full-length Trx cDNA from Korean rose bitterling (RuTrx). Multiple alignments of the deduced RuTrx polypeptide sequence and Trx homologs of other species were analyzed, and we conducted gene expression analysis of RuTrx transcripts during the early development of Korean rose bitterling and in various tissues from healthy fish. Moreover, the biological activity of the RuTrx was characterized following overexpression and purification of recombinant RuTrx protein. We demonstrated that recombinant RuTrx protein protected supercoiled DNA from oxidation-induced DNA cleavage *in vitro* by metal-catalyzed oxidation (MCO) assay. Dihydroethidium (DHE) staining for ROS production was examined in Raw264.7 cells to identify the antioxidant activity of recombinant RuTrx protein *in vivo*. This study is the first to report the molecular and functional analyses of the *R. uyekii* thioredoxin 1.

## 2. Results and Discussion

### 2.1. Analysis of R. uyekii Thioredoxin (RuTrx) the Nucleotide and Deduced Amino Acid Sequences

**Figure 1:**
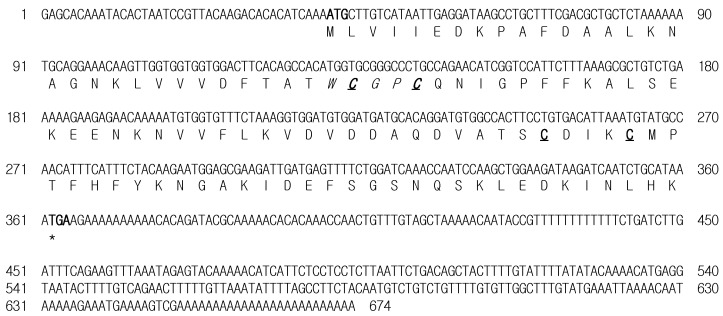
Nucleotide and deduced amino acid sequences of the *R. uyekii* thioredoxin (RuTrx). The start codon and stop codon are shown in bold. The active cysteine and two other cysteine residues are in bold and underlined, and the active site WCGPC is italicized.

The RuTrx full-length cDNA sequence was identified by expressed sequence tag (EST) analysis from a Korean rose bitterling *R. uyekii* cDNA library (GenBank accession no. KT279874). The nucleotide and deduced amino acid sequences are presented in Figure 1. The 674 bp RuTrx cDNA contains a 324 nt open reading frame (ORF) encoding a 107 aa protein preceded by a 40 nt 5′ UTR and followed by a 310 nt 3′ UTR with a poly (A) tail. The putative isoelectric point (pI) and molecular weight (*M*w) of the deduced protein of 107 aa were calculated as 5.28 and 12 kDa, respectively. There was no evidence of a signal sequence or an N-linked glycosylation site, indicating that the RuTrx protein might be a cytosolic form. In the deduced protein sequence, there was a characteristic redox active site, 31WCGPC35, and two other cysteine residues (Cys71 and Cys75). Two predicted disulfide bonds were identified (Cys32–Cys35, Cys71–Cys75). The cysteine residues found in the catalytic CGPC motif are essential for the activity of Trx. The CGPC motif is located on the surface of the protein in a short segment at the amino end of α-helix 2 [24].

**Figure 2:**
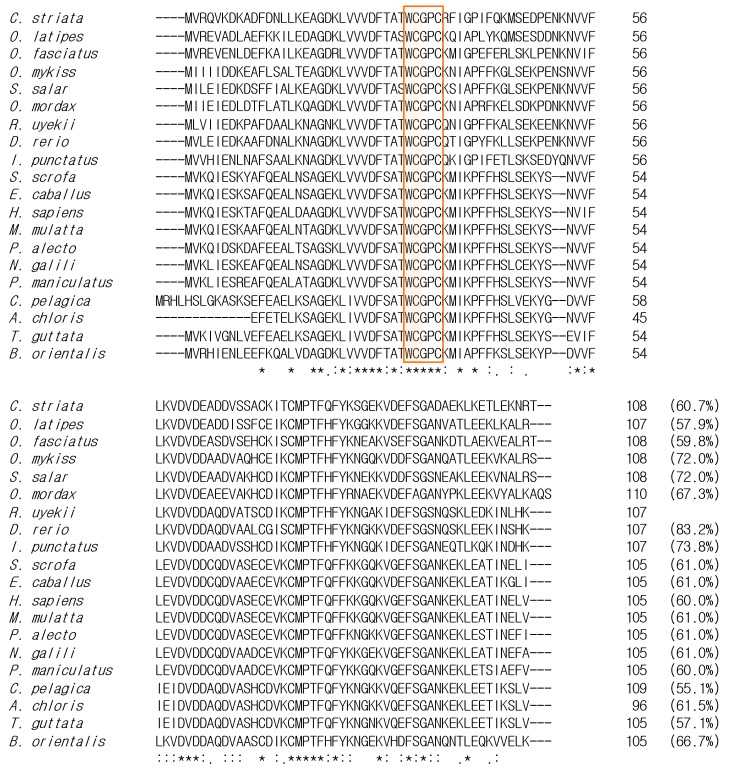
Multiple alignment of the deduced amino acid sequences of RuTrx with other species. Asterisk indicates identical residues at that position; colon denotes very similar residue at that position; dot indicates more or less similar, and no mark indicates no common property. Redox-active disulfide site, WCGPC, is boxed. The following sequences were extracted from GenBank: *Channa striata* (CCQ71730.1), *Oryzias latipes* (XP_004079889.1), *Oplegnathus fasciatus* (BAK38716.1), *Oncorhynchus mykiss* (ACO07759.1), *Salmo salar* (ACM09155.1), *Osmerus mordax* (ACO09971.1), *Danio rerio* (NP_001002461.1), *Ictalurus punctatus* (NP_001187021.1), *Sus scrofa* (NP_999478.1), *Equus caballus* (NP_001075282.1), *Homo sapiens* (CAA38410.1), *Macaca mulatta* (NP_001036197.2), *Pteropus alecto* (XP_006906153.1), *Nannospalax galili* (XP_008852851.1), *Peromyscus maniculatus* *bairdii* (XP_006985349.1), *Chaetura pelagica* (XP_010003764.1), *Acanthisitta chloris* (KFP72076.1), *Taeniopygia guttata* (NP_001232496.2) and *Bombina orientalis* (ACJ12082.1).

### 2.2. Comparison and Phylogenetic Analysis of RuTrx with Other Trx Homologs

**Figure 3:**
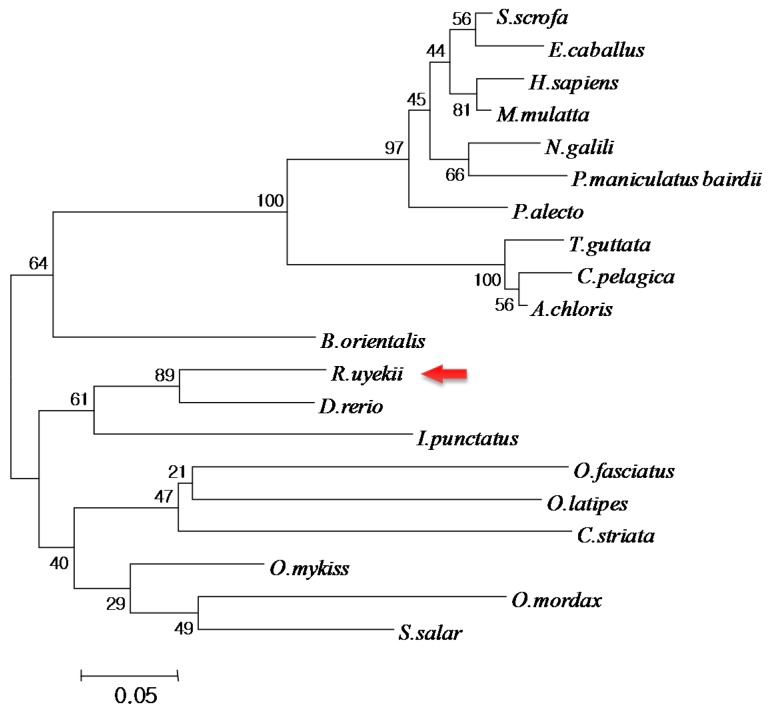
Phylogenetic analysis of the Korean rose bitterling RuTrx and related sequences. GenBank accession numbers for the analyzed sequences are: Snakehead murrel (*Channa striata*; CCQ71730.1), Asiatic ricefish (*Oryzias latipes*; XP_004079889.1), rock sea-bream (*Oplegnathus fasciatus*; BAK38716.1), redband trout (*Oncorhynchus mykiss*; ACO07759.1), Atlantic salmon (*Salmo salar*; ACM09155.1), rainbow smelt (*Osmerus mordax*; ACO09971.1), zebra fish (*Danio rerio*; NP_001002461.1), channel catfish (*Ictalurus punctatus*; NP_001187021.1), wild boar (*Sus scrofa*; NP_999478.1), horse (*Equus caballus*; NP_001075282.1), human (*Homo sapiens*; CAA38410.1), rhesus macaque (*Macaca mulatta*; NP_001036197.2), black flying fox (*Pteropus alecto*; XP_006906153.1), upper Galilee mountains blind mole rat (*Nannospalax galili*; XP_008852851.1), deer mouse (*Peromyscus maniculatus bairdii*; XP_006985349.1), chimney swift (*Chaetura pelagica*; XP_010003764.1), rifleman (*Acanthisitta chloris*; KFP72076.1), zebra finch (*Taeniopygia guttata*; NP_001232496.2) and oriental fire-bellied toad (*Bombina orientalis*; ACJ12082.1).

Pairwise alignment revealed the amino acid identity of RuTrx with orthologs, the highest being 83.2% with *Danio rerio*, followed by *Ictalurus punctatus* Trx (73.8%), *Oncorhynchus mykiss* Trx (72.0%), *Salmo salar* Trx (72.0%), *Osmerus mordax* Trx (67.3%), *Bombina orientalis* Trx (66.7%), *Acanthisitta chloris* Trx (61.5%), *Sus scrofa* Trx (61.0%), *Equus caballus* Trx (61.0%), *Macaca* *mulatta* Trx (61.0%), *Pteropus alecto* Trx (61.0%), *Nannospalax galili* Trx (61.0%), *Channa striata* Trx (60.7%), *Homo sapiens* Trx (60.0%), *Peromyscus maniculatus bairdii* Trx (60.0%), *Oplegnathus fasciatus* Trx (59.8%), *Oryzias latipes* Trx (57.9%), *Taeniopygia guttata* Trx (57.1%), and *Chaetura pelagica* Trx (55.1%) (Figure 2). Multiple sequence alignment revealed that the WCGPC redox active site was highly conserved among the Trx of all species examined. Including the species above, a variety of thioredoxin isoforms have been identified in organisms ranging from bacteria to human [24]. All thioredoxins identified to date possess a conserved CXXC motif at their redox active site [25]. To determine the evolutionary position of RuTrx, a phylogenetic tree was constructed using the neighbor-joining method (Figure 3). The Trx proteins from mammalia, amphibia, fish and bird clustered according to their corresponding subgroup with traditional taxonomy. RuTrx formed a cluster with Trx from zebrafish and channel catfish, and was phylogenetically separate from other species.

**Figure 4:**
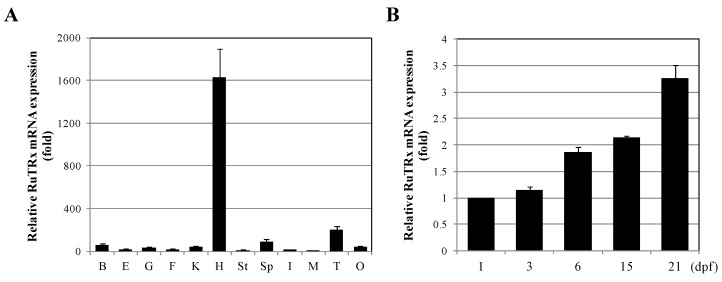
Expression of RuTrx mRNA in various tissues and during early development. (**A**) Quantitative real-time PCR analysis was performed using equal amounts of total RNA isolated from the Korean rose bitterling tissues. Relative mRNA levels were calculated using β-actin as an internal control. The mRNA level in each tissue was compared to that in the muscle, which was given an arbitrary value of 1. B, brain; E, eye; G, gill; F, fin; K, kidney; H, hepatopancreas; St, stomach; Sp, spleen; I, intestine; M, muscle; T, testis; O, ovary; (**B**) Quantitative real-time PCR analysis was performed using equal amounts of total RNA at 1, 3, 6, 15 and 21 days post-fertilization (dpf). The *C*_t_ values of RuTrx were used as absolute values. The expression levels were calculated relative to that of RuTrx mRNA at 1 dpf. All data are expressed as means ± SD (*n* = 3). Statistical significance was determined using the unpaired two-tailed Student’s *t*-test.

### 2.3. Expression Analysis of RuTrx mRNA in Various Tissues and During Early Development

To evaluate the tissue distribution of RuTrx mRNA, quantitative real-time polymerase chain reaction (PCR) was performed on 12 tissues from normally conditioned Korean rose bitterling (Figure 4A). The RuTrx mRNA levels were quantified after normalization to β-actin as an internal reference gene. The relative tissue-specific expression was determined by comparing the amount of transcript detected in each tissue with that in muscle. RuTrx transcripts were detected in all of the tissues examined, and hepatopancreas tissue showed the highest level of expression. In the brain, gill, kidney, spleen, testis and ovary, RuTrx expression was moderate, whereas it was very low in the eye, fin, stomach, intestine and muscles. Recent studies reported that the expression of Trx was most abundant in liver or hepatopancreas in *Oplegnathus fasciatus* and *Venerupis philippinarum* [26,27]. The liver is the main metabolic center for ROS and major site for detoxification [28]. The enrichment of RuTrx mRNA in hepatopancreas indicated the important physiological function of Trx.

The expression levels of RuTrx mRNA during early development of Korean rose bitterling were determined by quantitative real-time PCR at 1, 3, 6, 15 and 21 days post-fertilization (dpf) (Figure 4B). RuTrx transcripts were detected from 1 dpf and increased moderately during early development until 21 dpf (3.25-fold increase from 1 to 21 dpf). The thioredoxin mRNA of *Neobenedenia melleni* is present in the three development stages examined, with the highest expression level in the adult stage [29]. The thioredoxin mRNA of *Trypanosoma brucei* is also detected during all developmental stages [30]. Previous and the present studies suggest that thioredoxin may involve in important physiological functions during development. In addition, the highest expression of RuTrx at 21 dpf and NmTrx expression at the adult stage suggest they are required to control potential oxidized stress at that stage of development.

**Figure 5:**
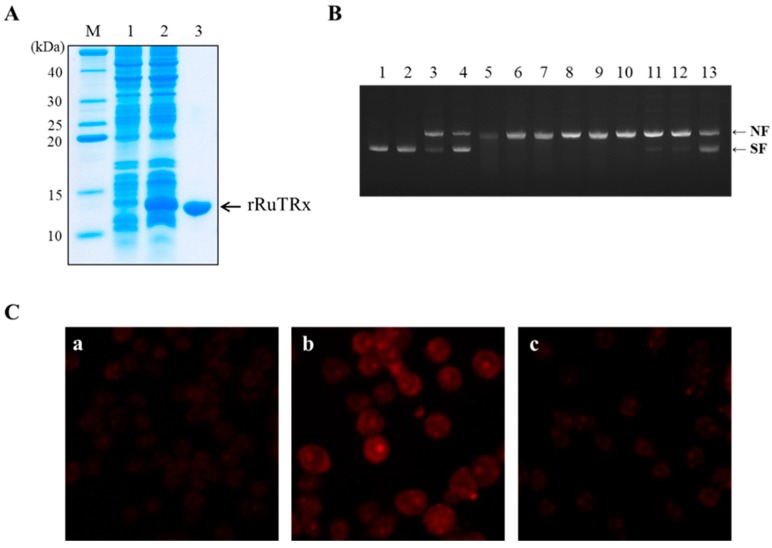
Expression and antioxidant activity of purified recombinant RuTrx protein under oxidative stress. (**A**) SDS-PAGE analysis of recombinant RuTrx protein. Protein samples were separated by 15% SDS-PAGE. Lane M, protein marker; Lane 1, non-induced pellet; Lane 2, 1 mM IPTG-induced supernatant; Lane 3, purified recombinant RuTrx protein; (**B**) Protective property of recombinant RuTrx protein against DNA cleavage from oxidative stress. Lane 1, pUC19 with no incubation; Lane 2, pUC19 and water; Lane 3, pUC19 and 20 μM FeCl_3_; Lane 4, pUC19, 2.5 mM DDT and water; Lane 5, pUC19, 20 μM FeCl_3_, 2.5 mM DDT and water; Lane 6, pUC19, 20 μM FeCl_3_, 2.5 mM DDT, water and BSA (50 μg/mL); Lane 7, pUC19, 20 μM FeCl_3_, 2.5 mM DDT, water and rRuTrx (50 μg/mL), Lane 8, pUC19, 20 μM FeCl_3_, 2.5 mM DDT, water and BSA (100 μg/mL); Lane 9, pUC19, 20 μM FeCl_3_, 2.5 mM DDT, water and rRuTrx (100 μg/mL); Lane 10, pUC19, 20 μM FeCl_3_, 2.5 mM DDT, water and BSA (200 μg/mL); Lane 11, pUC19, 20 μM FeCl_3_, 2.5 mM DDT, water and rRuTrx (200 μg/mL); Lane 12, pUC19, 20 μM FeCl_3_, 2.5 mM DDT, water and BSA (400 μg/mL); Lane 13, pUC19, 20 μM FeCl_3_, 2.5 mM DDT, water and rRuTrx (400 μg/mL). Samples in Lanes 2 to 13 were incubated for 1 h at 37 °C. NF, nicked form of pUC19; SF, supercoiled form of pUC19; (**C**) Effects of recombinant RuTrx protein on reducing ROS in Raw264.7 cells. DHE staining was performed to detect ROS in Raw264.7 cells after incubation with 10 μg/mL of rRuTrx for 24 h and treatment with H_2_O_2_ for 24 h. Fluorescence was detected under a microscope (Axiovert A1; Zeiss) with a 40× objective lens coupled to a digital camera. Representative fluorescence images of cells are shown: (**a**) vehicle-treated cells; (**b**) treated with 500 μM H_2_O_2_; (**c**) treated with 10 μg/mL rRuTrx and 500 μM H_2_O_2_. The red fluorescence intensity indicates the relative levels of ROS present in the cell.

### 2.4. Overexpression and Purification of Recombinant RuTrx Protein

The ORF region of RuTrx was cloned into pET22b(+) and highly expressed in soluble form in *E. coli* BL21 by IPTG-driven induction. SDS-PAGE analysis revealed a strong band with a molecular mass of approximately 12 kDa in induced cells (Lane 2) but not in non-induced cells (Lane 1) (Figure 5A). Recombinant RuTrx protein was purified from the induced cell culture and identified as a single band with a size of approximately 12 kDa.

### 2.5. Antioxidant Activity of Recombinant RuTrx Protein against DNA Cleavage in Vitro

Recombinant RuTrx protein (rRuTrx) was tested for its capacity to serve as an antioxidant enzyme using a metal-catalyzed oxidation (MCO) system (Figure 5B). Metal ions from FeCl_3_ generate ROS through the Fenton reaction, and the radicals produced by the MCO system cause DNA cleavage resulting in DNA nicking from supercoiled plasmid pUC19. Oxidation is one of the endogenous reactions that are likely to contribute to ongoing DNA damage [31]. The chemistry of DNA damage by several ROS has been well characterized *in vitro* [32,33]. Supercoiled DNA incubated without the MCO system was not damaged. However, with the MCO system, supercoiled DNA was totally nicked whereas supercoiled DNA incubated with the individual components of the MCO system (DDT, FeCl_3_) was slightly damaged. When rRuTrx was added to the MCO system, DNA nicking was averted (Figure 5B, Lanes 10–13). The ability of rRuTrx to protect DNA cleavage from oxidative nicking increased in a dose-dependent manner (Figure 5B, Lanes 11 and 13) indicating that rRuTrx protected supercoiled DNA from oxidation-induced DNA cleavage. Besides DNA, other biomolecules such as lipids and proteins are damaged by ROS [34] and the antioxidant activity of rRuTrx protein may have an inhibitory effect on damage to other biomolecules in cellular metabolism.

### 2.6. Antioxidant Activity of rRuTrx against ROS in Vivo

The specificity of DHE staining for ROS production was examined in Raw264.7 cells to identify the antioxidant activity of the rRuTrx (Figure 5C). In the presence of superoxide and other reactive species, DHE is oxidized to 2-hydroxyethidium and ethidium, which are trapped by intercalation with DNA resulting in bright red fluorescence [35]. Vehicle-treated cells showed basal levels of ROS. Compared to control cells, cells treated with 500 μM H_2_O_2_ exhibited enhanced ROS, which decreased with treatment of rRuTrx. The reduced ROS level demonstrated that rRuTrx inhibited ROS production against oxidative stress. Excess ROS induces undesirable cellular damage, and antioxidants such as Trx and glutathione are related to defense mechanisms against oxidative stress [36]. Besides, Trx has been found to possess a variety of biological functions, such as scavenging ROS, regeneration of oxidative-damage proteins, regulation of gene expression, controlling apoptosis as regulation of the NF-κB transcription factor, cell proliferation, and regulating signal transduction [15,26]. In addition, the expression of endogenous Trx is upregulated in response to oxidative stress and thioredoxin functions as a biomarker of oxidative stress [37], suggesting that RuTrx plays a key role in maintaining the redox state balance in Korean rose bitterling *R. uyekii*.

## 3. Experimental Section

### 3.1. Cloning of RuTrx from Rhoedeus uyekii

RuTrx cDNA was isolated through EST analysis of the Korean rose bitterling *R. uyekii* cDNA library (data not shown). EST clones were extracted from the *R. uyekii* cDNA library using a Plasmid Miniprep Kit (Qiagen, Seoul, Korea) and sequenced using T3 reverse primers (Promega, Madison, WI, USA) and an ABI3730xl automatic sequencer (Applied Biosystems, Inc., Oster City, CA, USA). The putative isoelectric point (pI) and the molecular weight (*M*w) of the deduced RuTRx protein were computed on the ExPASy Web site (http://web.expasy.org/compute_pi/). The signal sequence was found using SignalP (http://www.cbs.dtu.dk/services/SignalP/), and potential *N*-glycosylation site was assessed using the NetNGlyc1.0 Server (http://www.cbs.dtu.dk/services/NetNGlyc/). Disulfide bonds were predicted using DiANNA (http://clavius.bc.edu/~clotelab/DiANNA).

### 3.2. Multiple Sequence Alignment and Phylogenetic Analysis

Relevant sequences were compared using the BLASTX search program (http://www.ncbi.nlm.nih.gov/BLAST/). Multiple sequences of various species were retrieved from GenBank and aligned using CLUSTALW (http://www.genome.jp/tools-bin/clustalw). MEGA (ver. 4) was used to assess similarities among the aligned sequences. A phylogenetic tree based on the deduced amino acid sequences was constructed using a neighbor-joining algorithm, and the reliability of the branching was tested using bootstrap resampling with 1000 pseudo-replicates.

### 3.3. Quantitative Real-Time PCR

Total RNA was extracted from tissues using TRIzol™ reagent (Invitrogen, Carlsbad, CA, USA) according to the manufacturer’s instructions, treated with DNase I (New England BioLabs, Beverly, MA, USA) and quantitatively determined; 500 ng samples were used for reverse transcription (RT). First-strand cDNA was synthesized using the Transcriptor First Strand cDNA Synthesis Kit (Roche, Basel, Switzerland). Quantitative real-time PCR was performed using Fast SYBR^®^ Green Master Mix (Applied Biosystems, Inc.) and the following forward and reverse primers: RuTrx, RuTrx-RT-F (5′-TGG ACT TCA CAG CCA CAT GGT-3′) and RuTrx-RT-R (5′-CTT CTT TTT CAG ACA GCG CTT TAA-3′). Following an initial 10 min Taq activation step at 95 °C, real-time PCR was performed using 40 cycles of 95 °C for 10 s, 60 °C for 15 s, and fluorescence reading in an SDS 7500 system (Applied Biosystems, Inc.). All data are expressed as means ± SD (*n* = 3). Statistical significance was determined using the unpaired two-tailed Student’s *t*-test.

### 3.4. Recombinant RuTrx Plasmid Construction

To amplify the coding sequence of RuTrx, specific primers were designed with the *Nde*I restriction enzyme site at the N-terminus (RuTrx-NdeI-F, 5′-GCG CAT ATG ATG CTT GTC ATA ATT GAG-3′) and that of *Xho*I at the C-terminus (RuTrx-XhoI-R, 5′-CCG CTC GAG TTT ATG CAG ATT GAT CTT-3′). The PCR fragment and pET22b(+) vector (Novagen, Madison, WI, USA) were digested with both restriction enzymes and ligated. The recombinant clone was verified by DNA sequencing and then transformed into *E. coli* BL21 for protein expression.

### 3.5. Expression and Purification of Recombinant RuTrx Protein

The recombinant RuTrx protein was expressed with an N-terminal His tag in *E. coli*. Transformed *E*. *coli* was incubated at 37 °C with shaking at 200 rpm. When the optical density at 600 nm reached 0.5, isopropyl-β-thiogalactopyranoside (IPTG) was added to a final concentration of 1 mM for 3 h. Then, the cells were harvested by centrifugation for 30 min at 3000 rpm and stored at −80 °C. The cells were sonicated and the supernatant was collected for purification of the recombinant protein. The soluble proteins were loaded onto a nickel-nitrilotriacetic acid (Ni-NTA) column (Novagen). After washing with 60 mM imidazole, 500 mM NaCl, and 50 mM Tris–HCl buffer (pH 7.9), the bound protein was eluted using 1000 mM imidazole, 500 mM NaCl, and 20 mM Tris–HCl buffer (pH 7.9). Samples were collected at each stage of the purification process and separated by 15% SDS-PAGE with standard protein size markers (Life Technologies, Carlsbad, CA, USA). Protein bands were visualized using Coomassie blue G-250 stain, followed by destaining.

### 3.6. Animal and Tissue Preparation

*R. uyekii* were collected from the Yangchun River, Uiryung-gun, Gyungnam, Korea. The fish were maintained at the National Fisheries Research and Development Institute (NFRDI) in Busan, Korea. The adults were maintained in 40 L glass aquaria at a density of approximately 20 fish per aquarium. The water was renewed weekly and the temperature in the rearing tanks was maintained at 20 ± 1 °C. The room was maintained on a 12 h light:12 h dark cycle. Adults were fed TetraBits (Tetra, Melle, Germany) and frozen bloodworms (Advanced Hatchery Technology, Salt Lake City, UT, USA) twice per day. For RNA extraction, tissues were removed from three *R. uyekii* (mean body weight: 0.75 ± 0.3 g; mean total length: 4.0 ± 0.5 cm), immediately frozen in liquid nitrogen, and stored separately at −80 °C before use.

### 3.7. DNA Cleavage Assay

The ability of rRuTRx to protect against DNA cleavage due to oxidative nicking was evaluated using a metal-catalyzed oxidation (MCO) system. Each 50 μL reaction contained 20 μM FeCl_3_, 2.5 mM DDT and various concentrations of rRuTRx (50 to 400 μg/mL) or BSA as a negative control. Each reaction was incubated for 1 h at 37 °C following the addition of 1 μg of pUC19 supercoiled DNA. DNA bands were evaluated on a 1% agarose gel.

### 3.8. Cell Culture

Raw264.7 cells (American Tissue Culture Collection; ATCC, Manassas, VA, USA) were maintained in Dulbecco’s Modified Eagle Medium (DMEM; Welgene, Gyeongsan, Korea) with 10% heat-inactivated fetal bovine serum (FBS; Gibco-BRL, Gaithersburg, MD, USA) and 1% (*v*/*v*) antibiotic-antimycotic (AA; Gibco-BRL) at 37 °C in a humid atmosphere containing 5% CO_2_.

### 3.9. Detection of ROS

ROS, specifically superoxide, was assayed using the oxidative fluorescent dye, dihydroethidium (DHE; Invitrogen). Cells were seeded in a 96-well culture plate and treated with 10 μg/mL rRuTRx for 24 h. The medium was removed and replaced with cell culture medium containing 500 μM H_2_O_2_. Following 24 h incubation with H_2_O_2_, DHE was added to a final concentration of 10 μM for 20 min at 37 °C. Fluorescence was detected under a microscope (Axiovert A1; Zeiss, Oberkochen, Germany) with a 40× objective lens coupled to a digital camera.

## 4. Conclusions

In this study, the full-length cDNA encoding thioredoxin from Korean rose bitterling, *Rhodeus uyekii* was identified and characterized. The deduced RuTrx amino acid sequence showed a highly conserved characteristic redox active site, the CGPC motif. RuTrx transcripts were observed in all tissues examined, and hepatopancreas tissue showed the highest level of expression. In addition, recombinant RuTrx protein displayed the capacity to protect DNA from oxidation-induced DNA cleavage and antioxidant activity against ROS. Together, these results suggest that RuTrx may play a role in maintaining the redox state balance in Korean rose bitterling *R. uyekii*.
